# PoDPBT, a BAHD acyltransferase, catalyses the benzoylation in paeoniflorin biosynthesis in *Paeonia ostii*

**DOI:** 10.1111/pbi.13947

**Published:** 2022-10-27

**Authors:** Xiao‐Xiao Zhang, Jia‐Qi Zuo, Yi‐Ting Wang, Hui‐Yun Duan, Ming‐Hui Zhou, Hao‐Jie Li, Yong‐Hong Hu, Jun‐Hui Yuan

**Affiliations:** ^1^ College of Landscape Architecture and Arts Northwest A&F University Yangling China; ^2^ Shanghai Key Laboratory of Plant Functional Genomics and Resources Shanghai Chenshan Botanical Garden Shanghai China; ^3^ School of Ecological Technology and Engineering Shanghai Institute of Technology Shanghai China; ^4^ Shanghai Tenth People's Hospital Tongji University School of Medicine Shanghai China

**Keywords:** *O*‐benzoyltransferase, paeoniflorin biosynthesis, monoterpene, *Paeonia ostii*, BAHD acyltransferase

## Abstract

PoDPBT, an *O*‐benzoyltransferase belonging to the BAHD family, can catalyze the benzoylation of 8‐debenzoylpaeoniflorin to paeoniflorin. PoDPBT is the first enzyme demonstrated to be involved in the modification stage of paeoniflorin biosynthesis. DFGGG, a new DFGWG‐like motif, was revealed in the BAHD family. The transcriptome database provides a resource for further investigation of other enzyme genes involved in paeoniflorin biosynthesis.

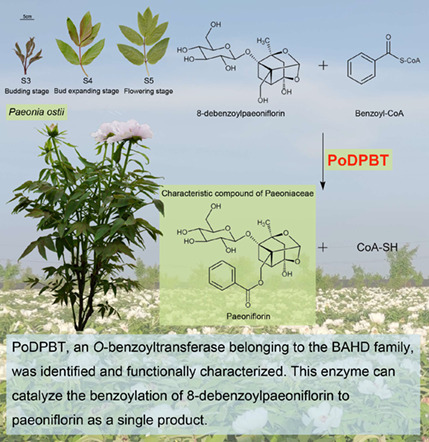

Paeoniflorin, a monoterpene bicyclic glycoside, is a very important medicinal compound with multiple pharmacological activities such as antidepression, antithrombosis, immunoregulation, and neuroprotection (Zhang *et al*., 2022; Zhao *et al*., 2020). Moreover, paeoniflorin is only found in Paeoniaceae plants and is considered as the characteristic compound of Paeoniaceae (Zhang *et al*., 2019). However, its biosynthesis pathway remains to be fully elucidated. Benzoyl‐CoA: benzyl alcohol *O*‐benzoyltransferases (BEBTs) belong to the BAHD acyltransferase family, and these enzymes possess a high affinity for benzoyl‐CoA as the acyl donor and can utilize different aromatic alcohols as the acyl acceptors (Chedgy *et al*., 2015). 8‐Debenzoylpaeoniflorin, one of derivatives of paeoniflorin, is also widely distributed in Paeoniaceae. Unlike paeoniflorin, 8‐debenzoylpaeoniflorin has a hydroxyl rather than a benzoyl group at the C8 position, and thus its structure is similar to the aromatic alcohol. Therefore, we speculated that an *O*‐benzoyltransferase existing in Paeoniaceae plants could utilize benzoyl‐CoA and 8‐debenzoylpaeoniflorin as substrates to synthesize paeoniflorin (Zhang *et al*., 2022). This enzyme would be designated as a benzoyl‐CoA: 8‐debenzoylpaeoniflorin 8‐*O*‐benzoyltransferase (DPBT).

We previously found the paeoniflorin contents in the leaf of *Paeonia ostii* were significantly correlated with the plant developmental stages (Zhang *et al*., 2019). To screen the genes encoding *O*‐benzoyltransferase involved in paeoniflorin biosynthesis, RNA samples from leaves were individually harvested from the budding stage (S3), bud expanding stage (S4) and flowering stage (S5) of *P. ostii* (Figure S1), and then sequenced on a BGISEQ‐500 platform (Figure 1a). The reliability of RNA‐Seq data was verified by qRT‐PCR (Figure S2 and Table S1). Twenty‐three *O*‐benzoyltransferase gene candidates were identified based on the gene annotations (Table S2) with different expression patterns at different development stages (Figure 1b). Among them, the expression levels of *Pos.gene26002*, *Pos.gene30573*, *Pos.gene35667*, *Pos.gene79215*, and *Pos.gene81370* were highly correlated (coefficient >0.9) with the paeoniflorin content. However, except for *Pos.gene30573*, the other four genes had the FPKM values below 1.00 across all the three development stages (Table S2). Therefore, we focused on the role of *Pos.gene30573* in converting 8‐debenzoylpaeoniflorin to paeoniflorin and named it as *PoDPBT*.

**Figure 1:**
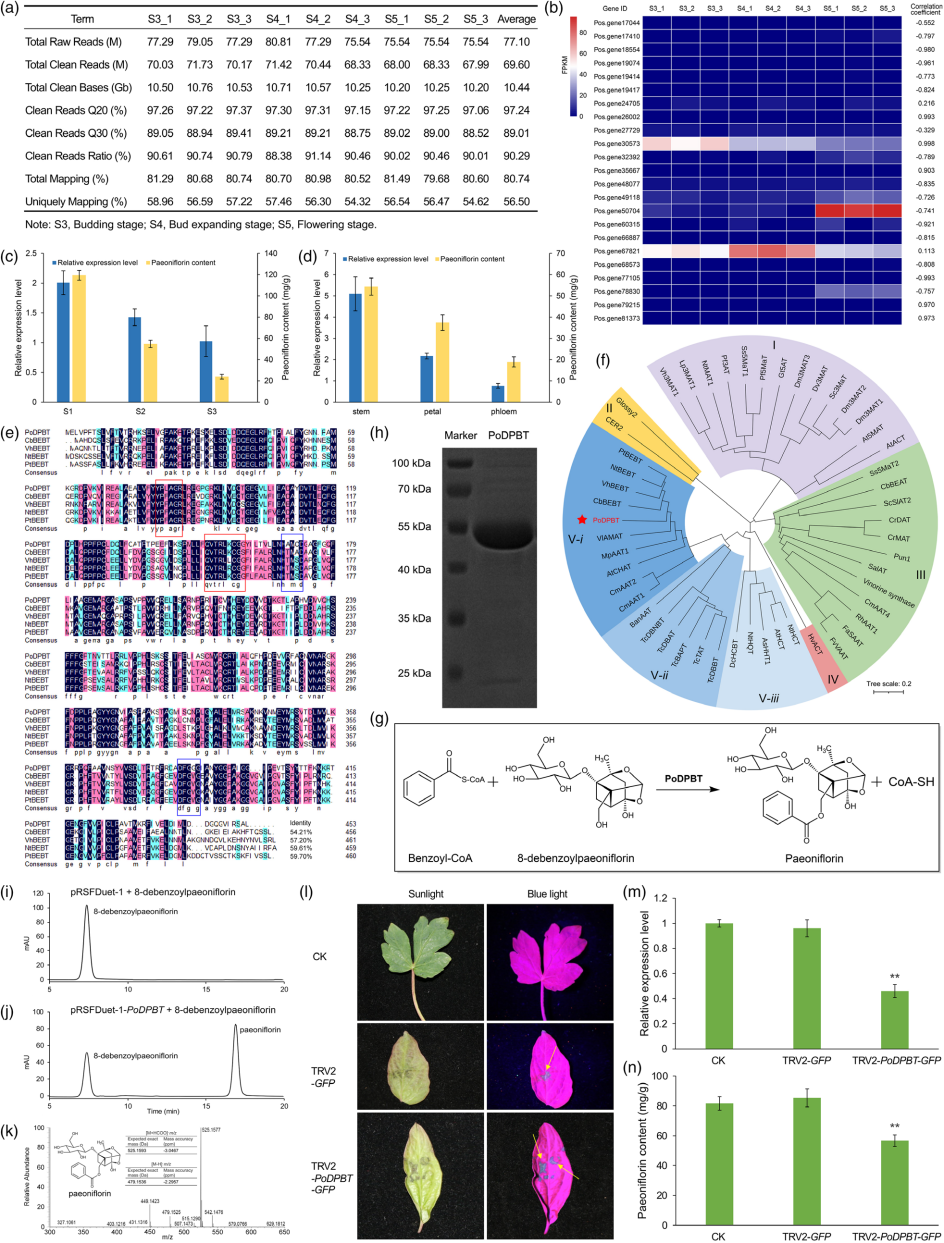
PoDPBT catalyses the benzoylation in the paeoniflorin biosynthesis. (a) Information of RNA‐seq raw data. (b) Expression levels of the 23 benzoyltransferase gene candidates in leaves of three developmental stages of *P. ostii* and their correlation with paeoniflorin content. (c, d) Expression levels of *PoDPBT* and paeoniflorin contents in different developmental stages (c) and organs (d). (e) Multiple sequence alignment of PoDPBT with BEBTs. Blue boxes show the HXXXD and DFGGG motif. Red boxes show the YPLAGR and QVTRLKCGG motif. (f) Neighbour‐joining phylogeny of PoDPBT and characterized BAHD members. (g) The reaction catalysed by PoDPBT. (h) SDS‐PAGE analysis of purified protein. (i–k) Characterization of PoDPBT by *in vitro* assay. UPLC profile of the product generated by empty vector enzyme with 8‐debenzoylpaeoniflorin (i) and the product of PoDPBT incubated with 8‐debenzoylpaeoniflorin (j); Mass spectrum data of the product of PoDPBT enzyme assay (k). (l–n) Silencing effect of *PoDPBT* on paeoniflorin production. Image of *P. ostii* leaves infected with TRV2‐*GFP* and TRV2‐*PoDPBT*‐*GFP* at 6 days after infiltration under blue light, and with normal plants as control (CK) (l); Expression levels of *PoDPBT* in different treatment leaves by qRT‐PCR (m); Paeoniflorin contents in different treatment leaves (n). Asterisks indicate statistically significant differences (**P < 0.01, Student's *t*‐test).

We then examined the expression pattern of *PoDPBT* in different leaf development stages of *P. ostii* by qRT‐PCR. *PoDPBT* was highest expressed in S3, followed by lower levels in S4 and S5, which was consistent with the accumulation of paeoniflorin (Figure 1c). Our previous study indicated paeoniflorin levels varied in different *P. ostii* organs (Zhang *et al*., 2019). The expression patterns were also checked, and the results showed the expression of *PoDPBT* was highest in the stem containing the most paeoniflorin (Figure 1d). These results further confirmed the correlation between the expression levels of *PoDPBT* and the paeoniflorin content, suggesting that *PoDPBT* is involved in the paeoniflorin biosynthesis.

Sequence analysis showed *PoDPBT* encodes a predicted 453‐amino acid protein of 49.91 kD. PoDPBT contains the HXXXD motif that is highly conserved for BAHD members, and another conserved region DFGWG motif is slightly altered with glycine instead of tryptophan. In addition to these two conserved motifs, YPLAGR and QVTRLKCGG motifs were also identified (Figure 1e) (Tuominen *et al*., 2011). These results indicated PoDPBT is a member of BAHD acyltransferase family. The phylogenetic tree showed PoDPBT is arranged in BAHD clade V‐*i* (Figure 1f), and members of this clade catalyse the formation of both volatile and non‐volatile esters (Chedgy *et al*., 2015). PoDPBT has a close relationship with CbBEBT, NtBEBT (D'Auria *et al*., 2002), VhBEBT (Togami *et al*., 2006), and PtBEBT (Chedgy *et al*., 2015). These results suggested PoDPBT could utilize benzoyl‐CoA and 8‐debenzoylpaeoniflorin to synthesize paeoniflorin (Figure 1g).

PoDPBT was expressed in *Escherichia coli*, and the purified protein size was consistent with the expected 49.91 kD (Figure 1h). *In vitro* enzyme assays were performed with benzoyl‐CoA and 8‐debenzoylpaeoniflorin. The UPLC‐MS results showed the new peak from experimental samples had the same retention time and MS spectrum as the authentic paeoniflorin standard (Figure 1i–k). These results confirmed PoDPBT acted as an *O*‐benzoyltransferase, which could convert 8‐debenzoylpaeoniflorin to paeoniflorin as a single product. To determine its affinity, kinetic analysis with different concentrations of 8‐debenzoylpaeoniflorin showed PoDPBT exhibited the Michaelis constant (*K*_m_) value of 1364.54 ± 166.28 μm, and the estimated catalytic constant (*K*_cat_) value of 0.54 ± 0.05 s^−1^.

To further validate the function of *PoDPBT in vivo*, we used a virus‐induced gene silencing system to suppress the expression of *PoDPBT* in *P. ostii* leaves. A tobacco rattle virus 2 (TRV2) vector was constructed with green fluorescent protein (GFP) as a reporter. Green fluorescent spots were observed under blue light in the leaves infiltrated with TRV2‐*GFP* and TRV2‐*PoDPBT*‐*GFP* (Figure 1l) but not in the normal plants. The expression level of *PoDPBT* in the leaves infiltrated with TRV2‐*PoDPBT*‐*GFP* was significantly lower than those of the normal plants and TRV2‐*GFP* lines, and there was no significant difference between the latter two lines (Figure 1m). The lowest content of paeoniflorin was also detected in the leaves of TRV2‐*PoDPBT*‐*GFP* lines (Figure 1n), and the remaining paeoniflorin is produced due to leftover PoDPBT. These results further confirmed the role of *PoDPBT* in the paeoniflorin biosynthesis.

Overall, PoDPBT, an *O*‐benzoyltransferase belonging to the BAHD family, was identified and functionally characterized, and this enzyme was able to catalyse the benzoylation of 8‐debenzoylpaeoniflorin to paeoniflorin as a single product. PoDPBT is the first enzyme involved in the modification stage of paeoniflorin biosynthesis. Other enzymes such as hydroxylase and glycosyltransferase involved in this stage have been predicted (Zhang *et al*., 2022), and these enzymes will be characterized successively based on the research strategy established in this study. These results will lay the foundation for the complete elucidation of paeoniflorin biosynthesis pathway.

## Conflicts of interest

The authors declare that they have no competing interest.

## Authors contributions

J.‐H.Y. and Y.‐H.H. conceived and designed the research. X.‐X.Z., J.‐Q.Z., Y.‐T.W., H.‐Y.D., M.‐H.Z., and H.‐J.L. performed the experiments. X.‐X.Z. and J.‐H.Y. analysed the data. X.‐X.Z. and J.‐H.Y. wrote the manuscript.

## Supporting information


**Figure S1** Leaves of *Paeonia ostii* at the budding stage (S3), bud expanding stage (S4) and flowering stage (S5).
**Figure S2** Analysis and validation of DEGs between the budding stage and flowering stage leaves.

**Table S1** The list of 19 DEGs validated by qRT‐PCR.
**Table S2** The list of 23 benzoyltransferase gene candidates.

**Appendix S1** Methods used in this study.
