# Supplementary material for: PoDPBT, a BAHD acyltransferase, catalyses the benzoylation in paeoniflorin biosynthesis in Paeonia ostii
Source: Plant Biotechnol J. 2022 Oct 27;21(1):14–6. doi: 10.1111/pbi.13947 (PMC9829388; doi:10.1111/pbi.13947)
Supplement: Supplementary file 1 — Figure S1 Leaves of Paeonia ostii at the budding stage (S3), bud expanding stage (S4) and flowering stage (S5). Figure S2 Analysis and validation of DEGs between the budding stage and flowering stage leaves. [file PBI-21-14-s002.doc]

**
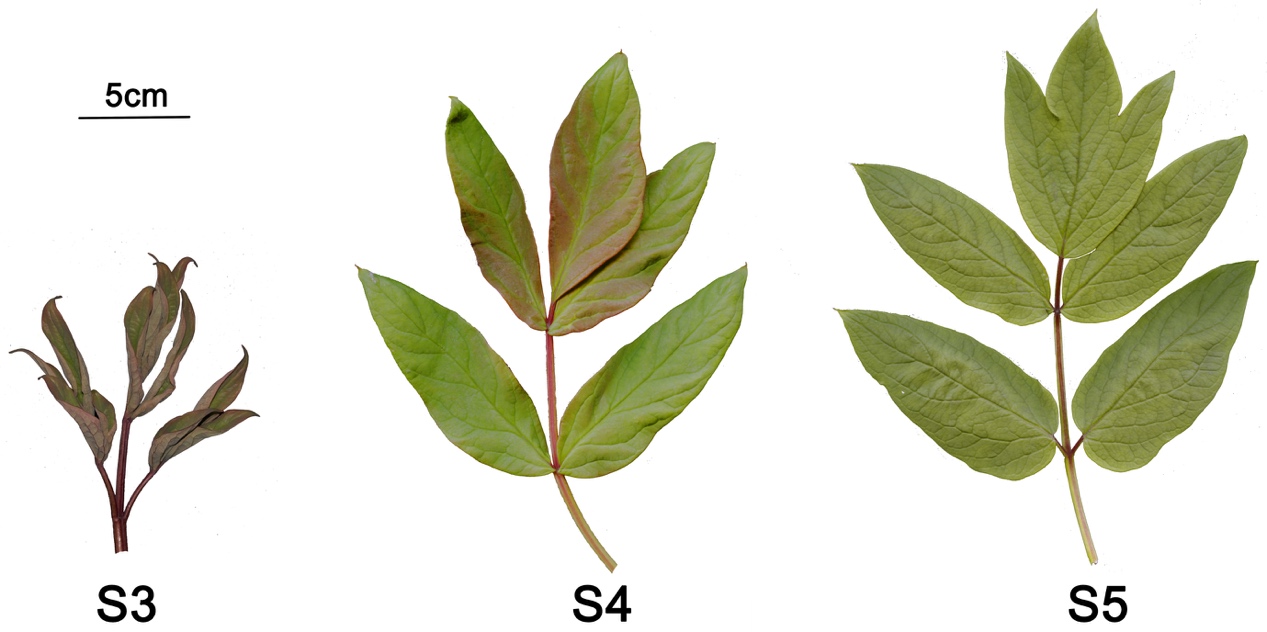
**

**Figure S1** The leaves of *Paeonia ostii* at the budding stage (S3), bud expanding stage (S4) and flowering stage (S5).

**
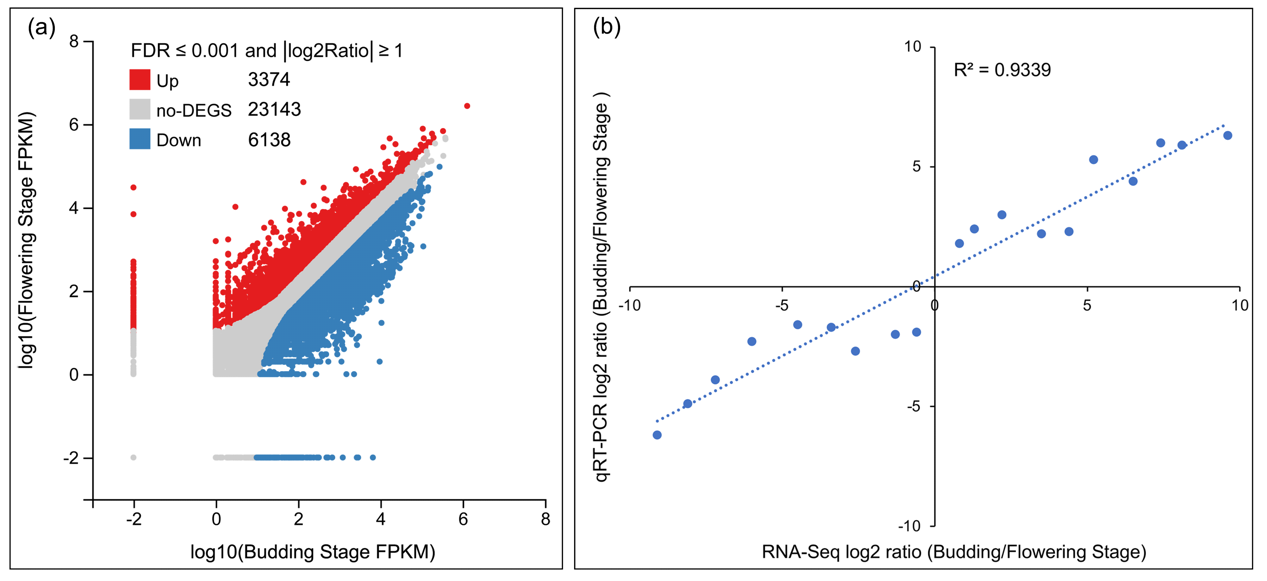
**

**Figure S2** Analysis and validation of differentially expressed genes (DEGs) between the budding stage and flowering stage leaves. (a) Scatter plot of DEGs. Red points represent upregulated DEGs, blue points represent downregulated DEGs, and gray points represent non-DEGs. False discovery rate (FDR) ≤0.001 and an absolute value of log2 ratio ≥1 were set as the threshold to judge the significance of gene expression differences. (b) Correlation of gene expression results obtained from RNA-Seq (*x*-axis) and qRT-PCR (*y*-axis) analysis.
