# Supplementary material for: PoDPBT, a BAHD acyltransferase, catalyses the benzoylation in paeoniflorin biosynthesis in Paeonia ostii
Source: Plant Biotechnol J. 2022 Oct 27;21(1):14–6. doi: 10.1111/pbi.13947 (PMC9829388; doi:10.1111/pbi.13947)
Supplement: Supplementary file 2 — Table S1 The list of 19 DEGs validated by qRT‐PCR. Table S2 The list of 23 benzoyltransferase gene candidates. [file PBI-21-14-s003.doc]

**Table S1 The list of 19 DEGs validated by qRT-PCR.**

| **Gene ID** | **S3 FPKM** | **S5 FPKM** | **log2(S5/S3)** | **S3_1 FPKM** | **S3_2 FPKM** | **S3_3 FPKM** | **S5_1 FPKM** | **S5_2 FPKM** | **S5_3 FPKM** |
| --- | --- | --- | --- | --- | --- | --- | --- | --- | --- |
| Pos.gene13781 | 112.10 | 0.19 | -9.25 | 137.28 | 102.54 | 96.47 | 0.06 | 0.50 | 0 |
| Pos.gene30069 | 82.96 | 0.22 | -8.51 | 131.55 | 0.21 | 117.13 | 0.49 | 0.09 | 0.08 |
| Pos.gene32266 | 294.23 | 1.65 | -7.49 | 234.96 | 357.68 | 290.06 | 0.25 | 2.74 | 1.95 |
| Pos.gene83675 | 103.23 | 1.28 | -6.36 | 90.19 | 134.87 | 84.63 | 0.37 | 2.94 | 0.54 |
| Pos.gene18200 | 159.93 | 3.04 | -5.87 | 119.44 | 169.35 | 191.01 | 1.22 | 7.14 | 0.75 |
| Pos.gene64442 | 373.99 | 13.75 | -4.75 | 549.13 | 98.39 | 474.44 | 16.15 | 16.40 | 8.70 |
| Pos.gene14900 | 576.65 | 41.89 | -3.80 | 660.34 | 600.29 | 469.33 | 26.35 | 85.99 | 13.34 |
| Pos.gene82005 | 1237.24 | 172.21 | -2.88 | 1294.40 | 1243.37 | 1173.96 | 73.17 | 363.00 | 80.47 |
| Pos.gene83474 | 1249.91 | 274.52 | -2.17 | 1098.45 | 1512.29 | 1138.99 | 245.55 | 332.88 | 245.14 |
| Pos.gene30573 | 55.17 | 22.46 | -1.28 | 67.76 | 39.14 | 58.62 | 28.13 | 19.89 | 19.37 |
| Pos.gene44067 | 177.87 | 369.45 | 1.06 | 186.04 | 157.30 | 190.26 | 406.91 | 271.06 | 430.39 |
| Pos.gene58900 | 91.24 | 343.15 | 1.90 | 89.19 | 79.95 | 104.58 | 276.33 | 507.86 | 245.25 |
| Pos.gene60727 | 58.04 | 374.99 | 2.70 | 70.38 | 37.62 | 66.11 | 306.58 | 384.60 | 433.80 |
| Pos.gene69643 | 42.46 | 509.19 | 3.58 | 21.90 | 57.36 | 48.13 | 331.82 | 788.01 | 407.73 |
| Pos.gene21335 | 15.79 | 264.60 | 4.06 | 11.16 | 12.18 | 24.04 | 155.54 | 474.01 | 164.26 |
| Pos.gene45817 | 34.70 | 1156.56 | 5.07 | 36.59 | 24.42 | 43.09 | 1147.92 | 1235.80 | 1085.97 |
| Pos.gene10821 | 1.73 | 116.72 | 6.09 | 1.67 | 1.15 | 2.37 | 114.28 | 86.81 | 149.06 |
| Pos.gene44505 | 0.45 | 89.98 | 7.64 | 0.57 | 0.42 | 0.37 | 108.73 | 47.48 | 113.72 |
| Pos.gene30902 | 0.81 | 246.86 | 8.28 | 0.64 | 0.65 | 1.15 | 213.11 | 257.68 | 269.79 |

Note: S3, Budding stage; S4, Bud expanding stage; S5, Flowering stage.

**Table S2 The list of 23 benzoyltransferase gene candidates.**

| **Gene ID** | **S3 FPKM** | **S4 FPKM** | **S5 FPKM** | **Kegg Orthology** | **Nr** | **GO** |
| --- | --- | --- | --- | --- | --- | --- |
| Pos.gene17044 | 0.01 | 0 | 0.05 | K19861//benzyl alcohol *O*-benzoyltransferase [EC:2.3.1.196 2.3.1.232] | XP_008232530.1|5.1e-152|PREDICTED: benzyl alcohol *O*-benzoyltransferase [*Prunus mume*] | \ |
| Pos.gene17410 | 0.08 | 0.34 | 3.06 | K19861//benzyl alcohol *O*-benzoyltransferase [EC:2.3.1.196 2.3.1.232] | CBI29212.3|2.7e-34|unnamed protein product, partial [*Vitis vinifera*] | GO:0016413//O-acetyltransferase activity;  GO:0016746//transferase activity, transferring acyl groups;  GO:0008374//O-acyltransferase activity;  GO:0016740//transferase activity;  GO:0016747//transferase activity, transferring acyl groups other than amino-acyl groups;  GO:0003824//catalytic activity;GO:0016407//acetyltransferase activity; |
| Pos.gene18554 | 0 | 0.06 | 0.11 | \ | XP_023924872.1|7.5e-13|anthranilate N-benzoyltransferase protein 1-like [*Quercus suber*] | GO:0016747//transferase activity, transferring acyl groups other than amino-acyl groups;  GO:0016746//transferase activity, transferring acyl groups;  GO:0016740//transferase activity;  GO:0003824//catalytic activity; |
| Pos.gene19074 | 0.13 | 0.42 | 0.81 | K19861//benzyl alcohol *O*-benzoyltransferase [EC:2.3.1.196 2.3.1.232] | XP_011012995.1|1.1e-147|PREDICTED: benzyl alcohol *O*-benzoyltransferase-like [*Populus euphratica*] | GO:0016746//transferase activity, transferring acyl groups;  GO:0016747//transferase activity, transferring acyl groups other than amino-acyl groups;  GO:0016740//transferase activity;  GO:0003824//catalytic activity; |
| Pos.gene19414 | 0 | 0.03 | 0.78 | K19861//benzyl alcohol *O*-benzoyltransferase [EC:2.3.1.196 2.3.1.232] | XP_015899457.1|7.4e-178|benzyl alcohol *O*-benzoyltransferase-like [*Ziziphus jujuba*] | GO:0016740//transferase activity;  GO:0016747//transferase activity, transferring acyl groups other than amino-acyl groups;  GO:0003824//catalytic activity;  GO:0016746//transferase activity, transferring acyl groups; |
| Pos.gene19417 | 0.02 | 0.04 | 0.17 | K19861//benzyl alcohol *O*-benzoyltransferase [EC:2.3.1.196 2.3.1.232] | XP_015899457.1|1.6e-75|benzyl alcohol *O*-benzoyltransferase-like [*Ziziphus jujuba*] | GO:0003824//catalytic activity;  GO:0016740//transferase activity;  GO:0016747//transferase activity, transferring acyl groups other than amino-acyl groups;  GO:0016746//transferase activity, transferring acyl groups; |
| Pos.gene24705 | 3.25 | 3.41 | 3.13 | K19861//benzyl alcohol *O*-benzoyltransferase [EC:2.3.1.196 2.3.1.232] | EOY10279.1|1.6e-25|Benzoyl coenzyme A: Benzyl alcohol benzoyl transferase, putative [*Theobroma cacao*] | GO:0016413//O-acetyltransferase activity;  GO:0016740//transferase activity;  GO:0016746//transferase activity, transferring acyl groups;  GO:0016407//acetyltransferase activity;  GO:0016747//transferase activity, transferring acyl groups other than amino-acyl groups;  GO:0008374//O-acyltransferase activity;  GO:0050643//10-deacetylbaccatin III 10-O-acetyltransferase activity;  GO:0003824//catalytic activity; |
| Pos.gene26002 | 0.06 | 0.02 | 0.01 | K19861//benzyl alcohol *O*-benzoyltransferase [EC:2.3.1.196 2.3.1.232] | PON81395.1|1.8e-189|Transferase [*Trema orientale*] | GO:0016740//transferase activity;  GO:0016746//transferase activity, transferring acyl groups;  GO:0016747//transferase activity, transferring acyl groups other than amino-acyl groups;  GO:0003824//catalytic activity; |
| Pos.gene27729 | 3.43 | 4.41 | 3.57 | K20240//spermidine dicoumaroyl transferase [EC:2.3.1.249];  K19861//benzyl alcohol *O*-benzoyltransferase [EC:2.3.1.196 2.3.1.232] | XP_002320943.3|6.3e-176|acyl transferase 4 [*Populus trichocarpa*] | GO:0008374//O-acyltransferase activity;  GO:0016746//transferase activity, transferring acyl groups;  GO:0016747//transferase activity, transferring acyl groups other than amino-acyl groups;  GO:0016413//O-acetyltransferase activity;  GO:0016740//transferase activity;  GO:0003824//catalytic activity;  GO:0050643//10-deacetylbaccatin III 10-O-acetyltransferase activity;  GO:0016407//acetyltransferase activity; |
| Pos.gene30573 | 55.17 | 35.04 | 22.46 | K19861//benzyl alcohol *O*-benzoyltransferase [EC:2.3.1.196 2.3.1.232] | GAV70647.1|3.9e-160|Transferase domain-containing protein [*Cephalotus follicularis*] | GO:0016747//transferase activity, transferring acyl groups other than amino-acyl groups;  GO:0016746//transferase activity, transferring acyl groups;  GO:0016740//transferase activity;  GO:0003824//catalytic activity; |
| Pos.gene32392 | 0.80 | 1.43 | 9.74 | K19861//benzyl alcohol *O*-benzoyltransferase [EC:2.3.1.196 2.3.1.232] | XP_015899457.1|9.6e-175|benzyl alcohol *O*-benzoyltransferase-like [*Ziziphus jujuba*] | GO:0003824//catalytic activity;  GO:0016740//transferase activity;  GO:0016746//transferase activity, transferring acyl groups;  GO:0016747//transferase activity, transferring acyl groups other than amino-acyl groups; |
| Pos.gene35667 | 0.46 | 0.36 | 0.11 | K19861//benzyl alcohol *O*-benzoyltransferase [EC:2.3.1.196 2.3.1.232] | EEF38389.1|1.2e-24|transferase, putative [*Ricinus communis*] | GO:0016746//transferase activity, transferring acyl groups;  GO:0016407//acetyltransferase activity;  GO:0008374//O-acyltransferase activity;  GO:0050643//10-deacetylbaccatin III 10-O-acetyltransferase activity;  GO:0016413//O-acetyltransferase activity;  GO:0003824//catalytic activity;  GO:0016740//transferase activity;  GO:0016747//transferase activity, transferring acyl groups other than amino-acyl groups; |
| Pos.gene48077 | 3.49 | 3.66 | 4.57 | K13065//shikimate *O*-hydroxycinnamoyltransferase [EC:2.3.1.133]  +ko01100//Metabolic pathways+ko01110//Biosynthesis of secondary metabolites+ko00940//Phenylpropanoid biosynthesis  +ko00941//Flavonoid biosynthesis+ko00945//Stilbenoid, diarylheptanoid and gingerol biosynthesis | XP_023898691.1|1.4e-183|anthranilate *N*-benzoyltransferase protein 3 [*Quercus suber*] | GO:0016746//transferase activity, transferring acyl groups;  GO:0003824//catalytic activity;  GO:0016747//transferase activity, transferring acyl groups other than amino-acyl groups;  GO:0016740//transferase activity; |
| Pos.gene49118 | 6.78 | 6.36 | 17.57 | K19861//benzyl alcohol *O*-benzoyltransferase [EC:2.3.1.196 2.3.1.232];  K19861//benzyl alcohol *O*-benzoyltransferase [EC:2.3.1.196 2.3.1.232];  K19861//benzyl alcohol *O*-benzoyltransferase [EC:2.3.1.196 2.3.1.232];  K19861//benzyl alcohol *O*-benzoyltransferase [EC:2.3.1.196 2.3.1.232];  K19861//benzyl alcohol *O*-benzoyltransferase [EC:2.3.1.196 2.3.1.232] | XP_015899457.1|2.0e-156|benzyl alcohol *O*-benzoyltransferase-like [*Ziziphus jujuba*] | GO:0016746//transferase activity, transferring acyl groups;  GO:0016747//transferase activity, transferring acyl groups other than amino-acyl groups;  GO:0016740//transferase activity;  GO:0003824//catalytic activity; |
| Pos.gene50704 | 5.97 | 4.84 | 93.08 | K19861//benzyl alcohol *O*-benzoyltransferase [EC:2.3.1.196 2.3.1.232] | XP_009372579.1|2.4e-175|PREDICTED: benzyl alcohol *O*-benzoyltransferase-like [*Pyrus x bretschneideri*] | \ |
| Pos.gene60315 | 0.09 | 2.06 | 6.14 | K19861//benzyl alcohol *O*-benzoyltransferase [EC:2.3.1.196 2.3.1.232] | XP_008232530.1|5.1e-151|PREDICTED: benzyl alcohol *O*-benzoyltransferase [*Prunus mume*] | GO:0016747//transferase activity, transferring acyl groups other than amino-acyl groups;  GO:0016746//transferase activity, transferring acyl groups;  GO:0016740//transferase activity;  GO:0003824//catalytic activity; |
| Pos.gene66887 | 0 | 0.02 | 0.20 | K19861//benzyl alcohol *O*-benzoyltransferase [EC:2.3.1.196 2.3.1.232] | XP_015899457.1|1.3e-171|benzyl alcohol *O*-benzoyltransferase-like [*Ziziphus jujuba*] | GO:0016746//transferase activity, transferring acyl groups;  GO:0016747//transferase activity, transferring acyl groups other than amino-acyl groups;  GO:0003824//catalytic activity;  GO:0016740//transferase activity; |
| Pos.gene67821 | 49.51 | 77.04 | 36.72 | K19861//benzyl alcohol *O*-benzoyltransferase [EC:2.3.1.196 2.3.1.232] | XP_023917914.1|1.7e-137|benzyl alcohol *O*-benzoyltransferase-like [Quercus suber] | GO:0016747//transferase activity, transferring acyl groups other than amino-acyl groups;  GO:0016740//transferase activity;  GO:0003824//catalytic activity;  GO:0016746//transferase activity, transferring acyl groups; |
| Pos.gene68573 | 0 | 0.05 | 0.48 | K19861//benzyl alcohol *O*-benzoyltransferase [EC:2.3.1.196 2.3.1.232] | XP_015899457.1|4.6e-125|benzyl alcohol *O*-benzoyltransferase-like [*Ziziphus jujuba*] | GO:0003824//catalytic activity;  GO:0016747//transferase activity, transferring acyl groups other than amino-acyl groups;  GO:0016740//transferase activity;  GO:0016746//transferase activity, transferring acyl groups; |
| Pos.gene77105 | 0 | 0.28 | 0.36 | K19861//benzyl alcohol *O*-benzoyltransferase [EC:2.3.1.196 2.3.1.232] | XP_023927880.1|3.0e-30|methanol *O*-anthraniloyltransferase-like [*Quercus suber*] | GO:0003824//catalytic activity;  GO:0016740//transferase activity;  GO:0016746//transferase activity, transferring acyl groups;  GO:0016747//transferase activity, transferring acyl groups other than amino-acyl groups; |
| Pos.gene78830 | 0.64 | 0.92 | 19.05 | K19861//benzyl alcohol *O*-benzoyltransferase [EC:2.3.1.196 2.3.1.232] | XP_015899457.1|9.1e-219|benzyl alcohol *O*-benzoyltransferase-like [*Ziziphus jujuba*] | GO:0003824//catalytic activity;  GO:0016746//transferase activity, transferring acyl groups;  GO:0016747//transferase activity, transferring acyl groups other than amino-acyl groups;  GO:0016740//transferase activity; |
| Pos.gene79215 | 0.04 | 0.02 | 0 | K19861//benzyl alcohol *O*-benzoyltransferase [EC:2.3.1.196 2.3.1.232] | XP_015940074.1|3.6e-17|benzyl alcohol *O*-benzoyltransferase [*Arachis duranensis*] | GO:0016747//transferase activity, transferring acyl groups other than amino-acyl groups;  GO:0003824//catalytic activity;  GO:0016740//transferase activity;  GO:0016746//transferase activity, transferring acyl groups; |
| Pos.gene81373 | 0.14 | 0.08 | 0.01 | K19861//benzyl alcohol *O*-benzoyltransferase [EC:2.3.1.196 2.3.1.232] | XP_015899457.1|1.6e-164|benzyl alcohol *O*-benzoyltransferase-like [*Ziziphus jujuba*] | GO:0003824//catalytic activity;  GO:0016740//transferase activity;  GO:0016746//transferase activity, transferring acyl groups;  GO:0016747//transferase activity, transferring acyl groups other than amino-acyl groups. |

Note: S3, Budding stage; S4, Bud expanding stage; S5, Flowering stage.
