# Supplementary material for: PoDPBT, a BAHD acyltransferase, catalyses the benzoylation in paeoniflorin biosynthesis in Paeonia ostii
Source: Plant Biotechnol J. 2022 Oct 27;21(1):14–6. doi: 10.1111/pbi.13947 (PMC9829388; doi:10.1111/pbi.13947)
Supplement: Supplementary file 3 — Appendix S1 Methods used in this study. [file PBI-21-14-s001.docx]

**Supplementary methods**

**RNA sequencing and bioinformatics analysis**

Total RNA was individually extracted from leaves of the budding stage (S3), bud expanding stage (S4) and flowering stage (S5) using RNA Prep Pure Plant Plus kit (Tiangen Biotech Co. Ltd., Beijing, China). The quality and quantity of RNA were assessed using NanoDrop 2000 (Thermo, CA, USA) and Agilent Bioanalyzer 2100 (Agilent Technologies, CA, USA), respectively.

The library construction was performed by Beijing Genomic Institution (Wuhan, China). The mRNA was enriched by Oligo (dT)-attached magnetic beads. The target RNA was fragmented with fragment buffer, and then double-strand cDNA (dscDNA) was generated by random hexamer-primed reverse transcription. The dscDNA was subjected to repair with phosphate at the 5′ end and sticky ‘A’ at the 3′ end, and then the adaptor with a sticky ‘T’ at the 3′ end was ligated to the 3′ ends of the dscDNA. The ligation products were amplified using two specific PCR primers. Subsequently, the PCR products were denatured by heat, and the single-strand DNA was cyclized by splint oligo and DNA ligase. Finally, the cDNA libraries were sequenced on a BGISEQ-500 platform.

All the raw sequencing reads were filtered to remove the low-quality reads (adaptor sequences, reads with more than 5% ambiguous ‘N’ bases, and reads with more than 20% low-quality bases) by SOAPnuke (Li et al., 2008). The clean reads were stored in FASTQ format and mapped to *P. ostii* genome sequences by HISAT2 (Kim et al., 2015) and Bowtie2 (Langmead and Salzberg, 2012). The gene expression level was calculated by RSEM (Li and Dewey, 2011) and expressed as the FPKM value (Mortazavi et al., 2008). Differential expression analysis was performed using the DESeq2 (Love et al., 2014) with Q value ≤ 0.05. To identify the functional annotation of the genes, a BLASTx search (E-value < 0.00001) was performed against protein databases, including non-redundant protein sequences (Nr) database and Kyoto Encyclopedia of Genes and Genomes (KEGG). Gene Ontology (GO) annotation was obtained using the Blast2GO program (E-value < 0.00001) (Conesa et al., 2005) based on NR annotation.

**qRT-PCR analysis**

Total RNA was extracted from the leaf, stem, petal and root as described above. First-strand cDNA was prepared from 1 μg of total RNA per sample, using a FastKing RT Kit with gDNase (Tiangen, Beijing, China). PCRs were performed on an ABI StepOnePlus^®^ Real-Time PCR System (Applied Biosystems, CA, USA), following the manufacturer’s instructions. Each reaction mixture (20 μl) contained 10 μl of TB Green Premix Ex Taq II (Tli RNaseH Plus) (Takara), 0.8 μl of each primer (10 μM), 0.3 μl of cDNA template (1 μg), and 8.1 μl of RNase-free water. PCR for each gene was performed in triplicate, with the following thermal cycling conditions: 95 °C for 30 s; 40 cycles of 95 °C for 5 s and 64 °C for 30 s; and 95 °C for 15 s. Primer specificity was confirmed via melt curve analysis. The relative expression levels of the tested genes were calculated via the 2^-ΔΔCt^ method, using the Actin (Pos.gene4533) gene as the internal control. Data are means ± standard deviation of three biological replicates. Gene-specific primers used in this study were listed in Table S3.

**Phylogenetic analysis**

Multiple Sequence Alignment was performed using DNAMAN 7 (Lynnon Biosoft, Quebec, Canada). The neighbor-joining tree was generated using MEGA X software (Kumar et al., 2018) with the following setting options: 1000 bootstrap replications, Poisson model, uniform rates and pairwise deletion. GenBank accession numbers of proteins used in the tree: Vh3MAT1, AAS77402; Lp3MAT1, AAS77404; NtMAT1, 2XR7_A; Pf3AT, Q9MBC1; Ss5MaT1, Q8W1W9; Pf5MaT, Q9LJB4; Gt5AT, Q9ZWR8; Dm3MAT3, BAF50706; Dv3MAT, Q8GSN8; Sc3MaT, AAO38058; Dm3MAT2, AAQ63616; Dm3MAT1, AAQ63615; At5MAT, Q9LJB4; AtACT, Q9FNP9; Ss5MaT1, Q8W1W9; CbBEAT, AAF04787; ScSlAT2, AFM77971; DAT, AAC99311; CrMAT, A8HYU5; Pun1, ADN97116; SalAT, Q94FT4; vinorine synthase, CAD89104; CmAAT4, AAW51126; RhAAT1, AAW31948; FaSAAT, AAG13130; FvVAAT, AX025504; HvACT, AAO73071; NtHCT, CAD47830; AtHCT, NP_199704; AsHHT1, BAC78633; NtHQT, CAE46932; DcHCBT, CAB06430; TcDBBT, Q9FPW3; TcTAT, AAF34254; TcBAPT, AAL92459; TcDBAT, AAF27621; TcDBNTBT, AAM75818; BanAAT, CAC09063; CmAAT1, CAA94432; CmAAT2, AAL77060; AtCHAT, AAN09797; MpAATI, AAU14879; VlAMAT, AAW22989; CbBEBT, AAN09796; VhBEBT, BAE72881; NtBEBT, AAN09798; PtBEBT, XP_002325454.2; Glossy2, DAA36076; Cer2, AAB17946.

**Protein expression and purification**

The full-length sequence of *PoDPBT* was obtained from the *P. ostii* reference genome. Specific forward and reverse primers were used to amplify the coding region of *PoDPBT*. The PCR products were digested by *Bam*H I and *Not* I, and then cloned into a hexahistidine-SUMO-tagged pRSFDuet-1 vector. The protein expression and purification were performed as described previously (Li et al., 2017). The purified protein was identified using SDS-PAGE analysis and stored in the buffer containing 20 mM Tris-pH 7.0, 500 mM NaCl and 5 mM dithiothreitol. The concentration was determined using the Bradford method with BSA as a standard (Bradford, 1976).

**Enzyme assays and kinetics**

*In vitro* enzyme assay was performed in a 40 μL reaction system consisting of 5 μL 8-debenzoylpaeoniflorin (20 mM), 5 μL benzoyl-CoA (50 mM), and 30 μL purified protein (0.64 mg/ml). The reaction was proceeded for 1 h at 30℃ and was stopped by flash-freezing in liquid nitrogen. The reaction product was extracted with 160 μL methanol and sonicated for 1 min. The mixture was filtered using a 0.22-mm filter and then analyzed by Ultra Performance Liquid Chromatography-Mass Spectrometry (UPLC-MS). The empty vector enzyme was used as a negative control.

For kinetics measurements, 8-debenzoylpaeoniflorin with a range from 0.5 to 20 mM was added to the reaction system above. The reaction time was 5 min. Michaelis constant (*K*_m_) and catalytic constant (*K*_cat_) values were evaluated using GraphPad Prism 7 software (GraphPad Software, Inc.). Data are means ± standard deviation of three biological replicates.

**UPLC-MS analysis**

Chromatographic analysis was performed using the Thermo Scientific Syncronis C18 column (100 mm × 2.1 mm, 1.7 µm) based on Dionex UltiMate 3000 UPLC system (Thermo Fisher Scientific, MA, USA). The mass spectrum was collected on a Q Exactive Plus hybrid quadrupole-Orbitrap mass spectrometer (Thermo Fisher Scientific, MA, USA). The detailed parameters were described in our previous study (Zhang et al., 2019). The standard curve was plotted using authentic paeoniflorin for quantification. All of the samples were tested in triplicate, and each sample was injected once. Data are means ± standard deviation.

**Virus-induced gene silencing assay**

The silencing of *PoDPBT* in leaves by virus-induced gene silencing was performed as previously described (Xie et al., 2019; Yang et al., 2021). A tobacco rattle virus 2 (TRV2) vector was constructed with green fluorescent protein (GFP) as a reporter. The TRV1 and TRV2-*GFP* plasmids were kept in our laboratory. To generate the TRV2-*PoDPBT*-*GFP* construct, a 332-bp *PoDPBT* fragment was PCR-amplified using specific primers (Table S3). All three TRV constructs were transformed into *Agrobacterium tumefaciens* strain GV3101, and then the transformed bacteria were cultured at 28°C with constant shaking at 220 r/min overnight. The bacterial cells were centrifuged at 4000 *g* and resuspended in infiltration buffer (10 mM MgCl_2_, 10 mM MES, and 200 μM acetosyringone) to a final OD600 of 1.0. The cultures containing TRV1 and TRV2 constructs were shaken lightly for 6 h at 28°C and mixed in a 1:1 ratio (*v*/*v*). For vacuum infiltration, whole plants were submerged in the infiltration buffer under a 0.8 MPa pressure for 20 min. The leaves of normal plants and plants infiltrated with TRV2-*GFP* were used as controls. At 6 d after infiltration, the leaves were collected for GFP analysis. At 10 d after infiltration, the leaves were collected for qRT-PCR and paeoniflorin quantification. Data are means ± standard deviation of three biological replicates.

**References**

Bradford MM (1976) A rapid and sensitive method for the quantitation of microgram quantities of protein utilizing the principle of protein-dye binding. *Anal. Biochem.* **72**: 248–254

Conesa A, Götz S, García-Gómez JM, Terol J, Talón M, Robles M (2005) Blast2GO: A universal tool for annotation, visualization and analysis in functional genomics research. *Bioinformatics* **21**: 3674–3676

Kim D, Langmead B, Salzberg SL (2015) HISAT: A fast spliced aligner with low memory requirements. *Nat. Methods* **12**: 357–360

Kumar S, Stecher G, Li M, Knyaz C, Tamura K (2018) MEGA X: Molecular evolutionary genetics analysis across computing platforms. *Mol. Biol. Evol.* **35**: 1547–1549

Langmead B, Salzberg SL (2012) Fast gapped-read alignment with Bowtie 2. *Nat. Methods* **9**: 357–359

Li B, Dewey CN (2011) RSEM: Accurate transcript quantification from RNA-Seq data with or without a reference genome. *BMC Bioinformatics* **12**: 323

Li H, Liefke R, Jiang J, Kurland JV, Tian W, Deng P, Zhang W, He Q, Patel DJ, Bulyk ML, et al (2017) Polycomb-like proteins link the PRC2 complex to CpG islands. *Nature* **549**: 287–291

Li R, Li Y, Kristiansen K, Wang J (2008) SOAP: Short oligonucleotide alignment program. *Bioinformatics* **24**: 713–714

Love MI, Huber W, Anders S (2014) Moderated estimation of fold change and dispersion for RNA-seq data with DESeq2. *Genome Biol.* **15**: 550

Mortazavi A, Williams BA, McCue K, Schaeffer L, Wold B (2008) Mapping and quantifying mammalian transcriptomes by RNA-Seq. *Nat. Methods* **5**: 621–628

Xie L, Zhang Q, Sun D, Yang W, Hu J, Niu L, Zhang Y (2019) Virus-induced gene silencing in the perennial woody *Paeonia ostii*. *PeerJ* **7**: e7001

Zhang X, Zhai Y, Yuan J, Hu Y (2019) New insights into Paeoniaceae used as medicinal plants in China. *Sci. Rep.* **9**: 18469

**Table S3 Primers used in this study.**

| **Gene ID** | **Forward primer sequence** | **Reverse primer sequence** | **Purpose** |
| --- | --- | --- | --- |
| Pos.gene4533 | GCTGGGTACACAACATCTAA | AATATTTTCCCCCCGTTCCC | qRT-PCR |
| Pos.gene13781 | CTGACTTCTTGCCTTTCTTGA | ATTCCTTTCCATCGCCTTCAA |  |
| Pos.gene30069 | ACAATTGTCGTGAAGGTTCCG | CGTCTTCGATTTGGGTTTTGC |  |
| Pos.gene32266 | CTTATGATGCAACTGAGAACG | CTTGCCTTCTCCTTGATTTCA |  |
| Pos.gene83675 | CTTCCACAATTTCGCATGCGT | CATATGCAACTACGTGATCCT |  |
| Pos.gene18200 | AGCAACCAAGATAATCGGAGT | TTTTGTGGACTCTAAGGCTTC |  |
| Pos.gene64442 | ATTTGCTTACATCTGGGTTGG | AAGTAACCATTTCCAAACGGG |  |
| Pos.gene14900 | TTTACGAGTTGAGTGACACCA | GCAATAGTTCGGAGCTTCTT |  |
| Pos.gene82005 | CGTTGTTTCCTGCGCAGATAT | CCAATGGAAGCAAACCTCTCT |  |
| Pos.gene83474 | TAAGTTCAAGCTCTGCAAGGT | ACCAGTCACCATCACAACATT |  |
| Pos.gene44067 | TTCTCCTTCGATACATCCCAT | TGTCCAGTATGTCATCCCAGT |  |
| Pos.gene58900 | TAACGAAGAAAATGTGCTGGG | AGAGGAGGTTCATTTTGTGGT |  |
| Pos.gene60727 | CGTCCGGGGATCTAAATTTAT | CTACCATTTCCTCTGAGACAT |  |
| Pos.gene69643 | CCATACTAAAGAGGGTGTTGA | GTGGAGAATAGTGAAGAGCTT |  |
| Pos.gene21335 | TCCCCTTACACCTCAAATAGT | ATATCGTAATTCAATCGCCTG |  |
| Pos.gene45817 | TCTTCGCTCGTGATATGGTGA | TCACGTTAGTATTCCCACCTT |  |
| Pos.gene10821 | CATCATATTCTACGTCGACGA | GGTAAATTCAGCGATAAAGG |  |
| Pos.gene44505 | GCCCGGTATGTCTTCTTAAAA | TTTGGTGATGGTAGTGGAAGT |  |
| Pos.gene30902 | TCACATCTACAGTTGGCTTTG | CCTCATTTTCTTCTCTGCTTG |  |
| Pos.gene30573  (PoDPBT) | AAAGGTACACTAGCTCCCCAT | GCCACATGCAAGAAGCTATTA |  |
| PoDPBT | CATCACCACAGCCAGGATCCATGGAATTGGTACCTCCCACAT | ACTTAAGCATTATGCGGCCGCTTAGAGAGCAGATCTGATGACC | Cloning |
| TRV2-GFP-PoDPBT | GAAGGCCTCCATGGGGATCCCGAAACCCACCACGCATTACAT | GGACATGCCCGGGCCTCGAGGCAGCAGGAGAAGCAATCACAT | VIGS |
